# Systemic effect of catumaxomab in a patient with metastasized colorectal cancer: a case report

**DOI:** 10.1186/1471-2407-13-618

**Published:** 2013-12-31

**Authors:** Angelika Bezan, Florian Hohla, Thomas Meissnitzer, Richard Greil

**Affiliations:** 1 IIIrd Medical Department with Hematology, Medical Oncology, Hemostaseology, Rheumatology and Infectiology, Paracelsus Medical University of Salzburg, Müllner Hauptstrasse 48, 5020 Salzburg, Austria; 2 Institute of Radiology, Paracelsus Medical University of Salzburg, Müllner Hauptstrasse 48, 5020 Salzburg, Austria

**Keywords:** Immunotherapy, Catumaxomab, Systemic effect, Colorectal cancer, Ascites

## Abstract

**Background:**

Catumaxomab, the first anti-EpCAM antibody, was approved in 2009 for the treatment of malignant ascites in cancer patients with EpCAM positive tumors. We consider this case of interest as treatment with catumaxomab not only prolonged the puncture-free interval but also showed a systemic effect in a patient with metastasized colorectal cancer by regression of a pulmonary metastasis.

**Case presentation:**

We describe the case of a 78 year old patient initially diagnosed with locally advanced colon cancer and one synchronous liver metastasis in September 2010 who was treated by hemicolectomy and simultaneous atypical liver resection. During adjuvant chemotherapy the patient developed a peritoneal carcinomatosis with ascites in March 2011. Palliative chemotherapy was not well tolerated and therefore refused by the patient. Because of disease progression in June 2011 with one pulmonary metastasis and clinically predominant ascites an immunotherapy with intraperitoneal catumaxomab was started in December 2011. After treatment with catumaxomab a puncture free survival of 12 months as well as a regression of the pulmonary lesion was achieved until January 2013.

**Conclusion:**

This case suggests that treatment with catumaxomab does not only improve quality of life by local suppression of malignant ascites but also might have a systemic antitumor effect.

## Background

Metachronous peritoneal carcinomatosis (PC) has been reported in 4-12% of patients with colorectal cancer (CRC) [1]. According to the EVOCAPE 1 study median overall survival for patients with PC from CRC is 6.9 months, depending on TNM stage, size and distribution of peritoneal nodules and the presence of ascites [2]. Modern systemic chemotherapy as well as cytoreductive surgery and hyperthermic intraperitoneal (i.p.) chemotherapy are current treatment options for selected patients to prolong overall survival [3]. Malignant ascites, which is caused by the intraperitoneal spread of tumor cells, has an immense impact on the quality of life due to its symptoms and frequent recurrence [4]. Peritoneal tumor cells of CRC origin frequently overexpress the epithelial cell-adhesion molecule (EpCAM) [5]. Catumaxomab, which has been approved for the treatment of malignant ascites [6], has antigen-binding sites to epithelial tumor cells via EpCAM and to T-cells via CD3. With the Fc domain catumaxomab activates accessory cells resulting in a pro-inflammatory response leading to an immunologic antitumor effect [7].

We report on a 78 years old patient with peritoneal and pulmonary metastasized CRC. We consider this case of interest as treatment with i.p. Infusions of catumaxomab not only prolonged the puncture-free interval but also showed a systemic effect by regression of a pulmonary metastasis.

## Case presentation

In September 2010 a 78 years old man was diagnosed with an adenocarcinoma of the right-sided colon. Initial staging by abdominal and pulmonary CT revealed a lesion in the right-sided colon penetrating the visceral peritoneum with infiltration of the abdominal wall (cT4b) and a single metastatic lesion in the liver, in segment VII, with a diameter of 1.5 cm (M1a) according to a stage IVA disease.

**Figure 1:**
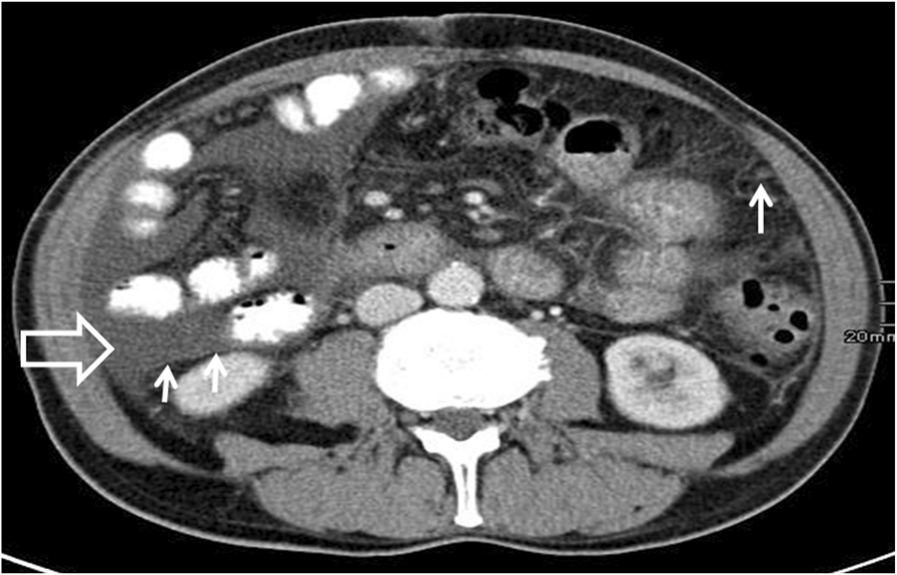
Contrast-enhanced computed tomography scan of the abdomen showing ascites (big arrow) and peritoneal nodules (small arrows) in March 2011.

After right-sided hemicolectomy together with a simultaneous atypical resection of the single liver metastasis adjuvant chemotherapy (CTX) with capecitabine and oxaliplatin was started. After 5 cycles of adjuvant CTX the patient complained about a considerable increase of his abdominal girth. An abdominal CT in March 2011 yielded peritoneal nodules and ascites (Figure 1).

After confirmation of a mutated K-RAS gene a palliative first line CTX with capecitabine and irinotecan in combination with bevacizumab was started. However, treatment was interrupted right after the first cycle because of an episode with neutropenic fever and resumption of CTX was refused by the patient until June 2011.

**Figure 2:**
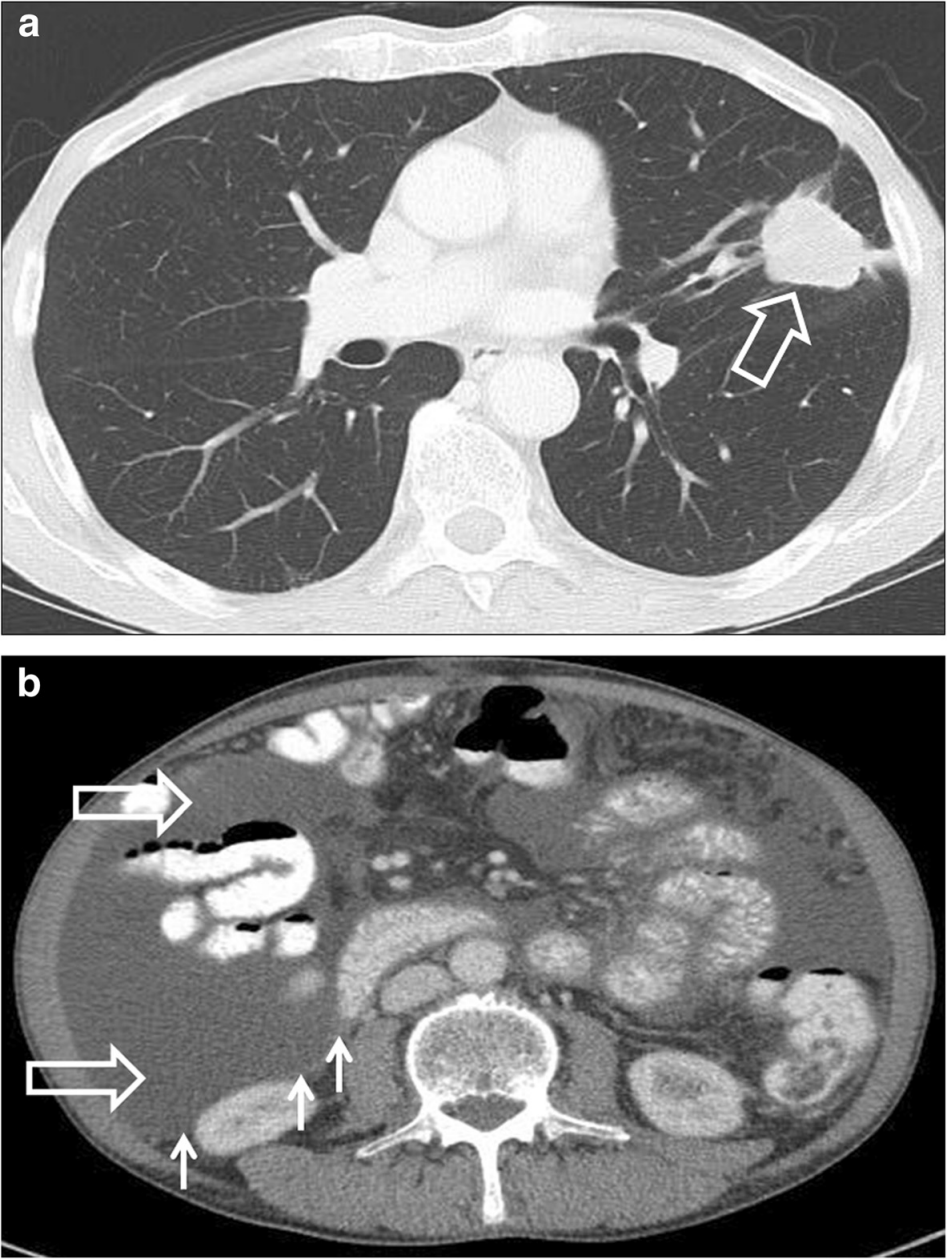
**Contrast-enhanced computed tomography scan of the chest and abdomen showing disease progression in June 2011. (a)** A 3.3 × 2.8 cm mass (big arrow) in the left lung and **(b)** ascites in all 4 quadrants of the abdomen (big arrow) and peritoneal nodules (small arrows).

**Figure 3:**
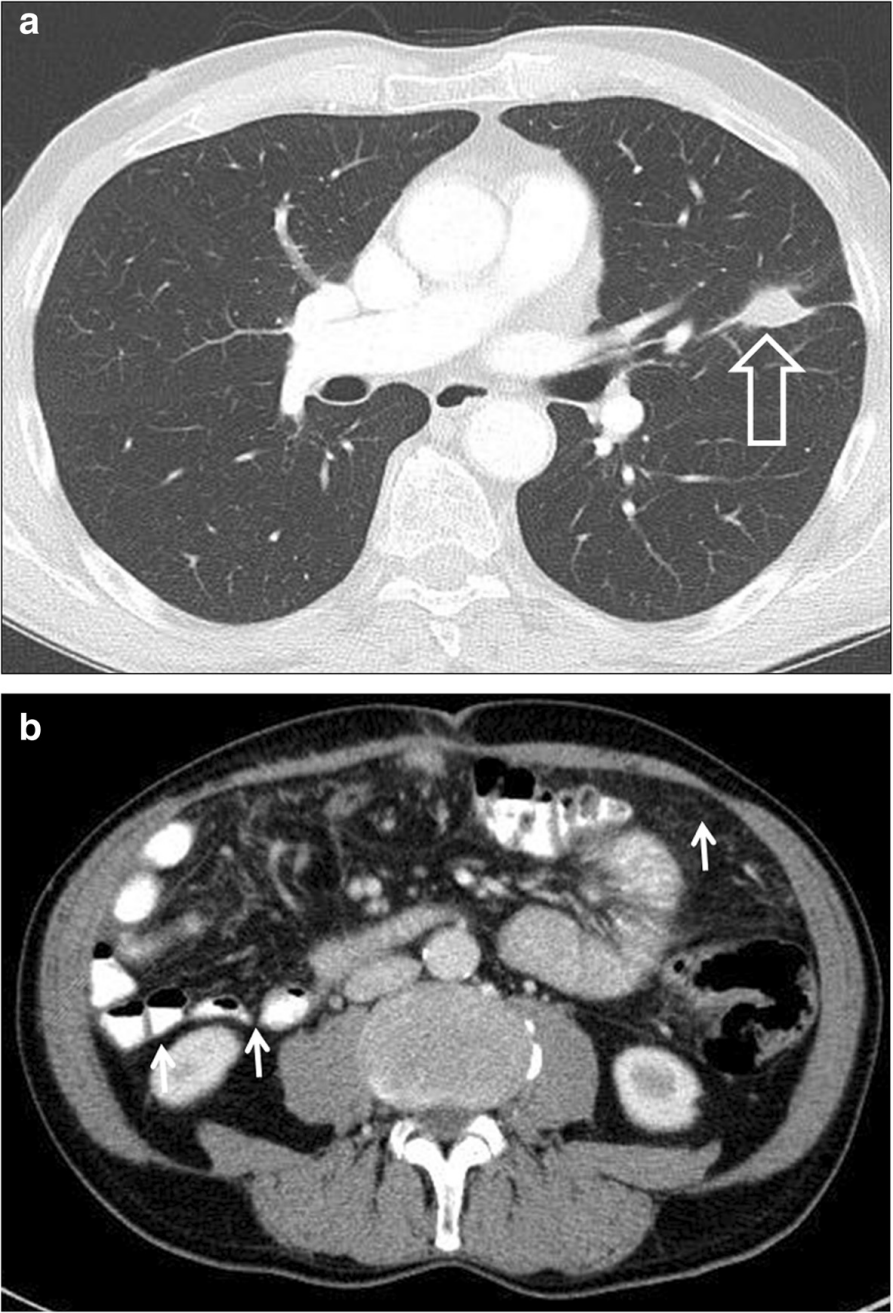
**Contrast-enhanced computed tomography scan of the chest and abdomen after treatment with catumaxomab in July 2012. (a)** Partial response of the pulmonary lesion (1.8 × 1.1 cm, big arrow) and **(b)** regression of peritoneal carcinomatosis (small arrows) with less amount of ascites.

**Figure 4:**
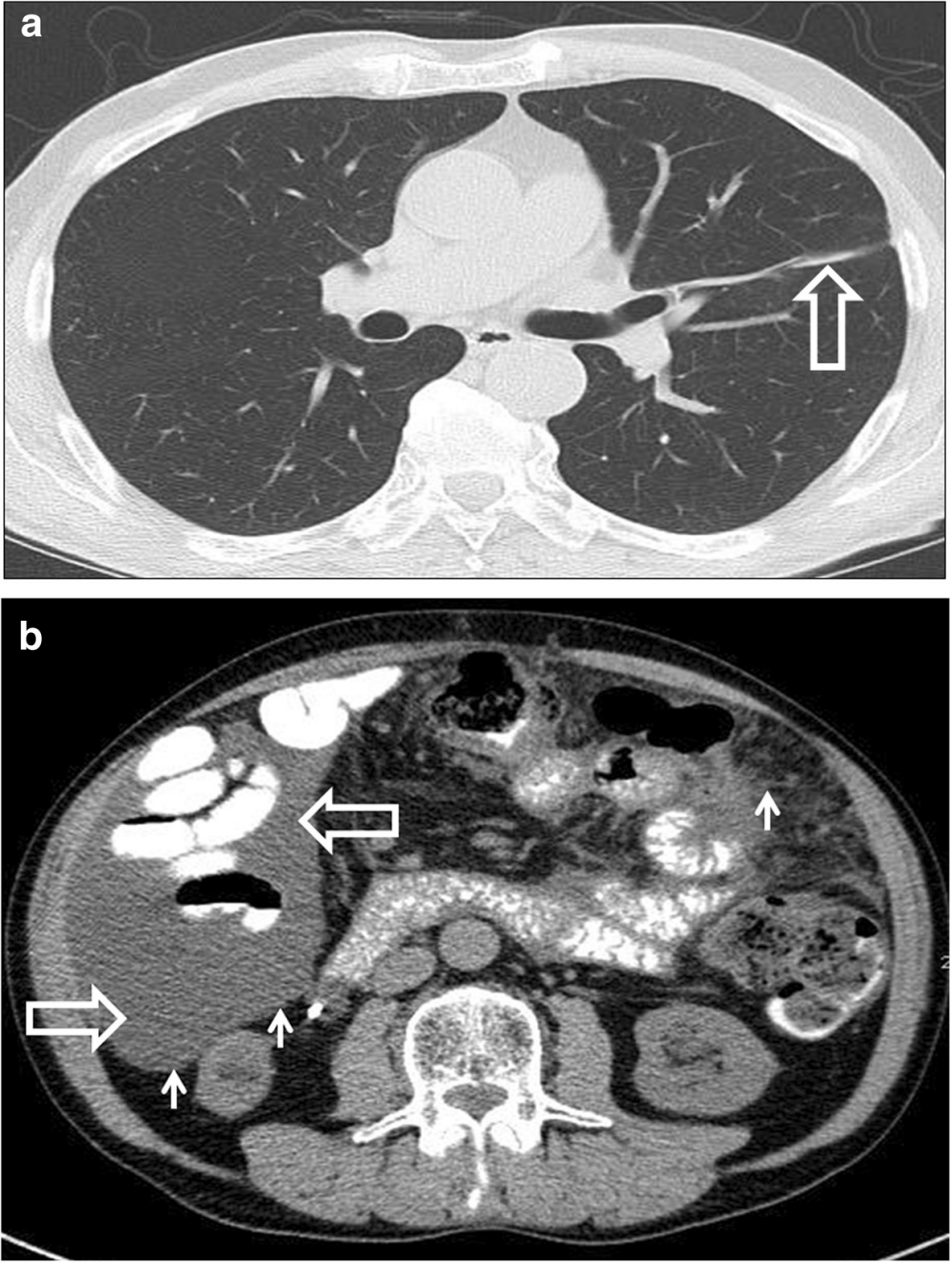
**Contrast-enhanced computed tomography scan of the chest and abdomen in January 2013. (a)** The pulmonary lesion can only be detected as a scar (big arrow). **(b)** Recurrent ascites (big arrows) and peritoneal nodules (small arrows).

CT imaging in June 2011 showed a disease progression with a new solitary pulmonary lesion with a diameter of 3.3 × 2.8 cm (Figure 2a) and ascites in all 4 quadrants of the abdomen (Figure 2b). As the patient refused biopsy of the pulmonary lesion and the radiology report stated that the lung lesion was radiological consistent with a pulmonary metastasis a biopsy was not performed. Tumor markers CEA and CA 19.9 were within normal range at the time of diagnosis and during the whole course of disease. Clinically, the amount of ascites was increasing and required abdominal punctures on a weekly basis. Therefore the first line CTX with capecitabine, irinotecan and bevacizumab was reinitiated. Despite administration of granulocyte colony stimulation factor another episode of neutropenic fever occurred and CTX was discontinued after a total of 3 cycles in August 2011. As the patient refused to receive any further CTX and because of ascites being the predominant clinical symptom an immunotherapy with i.p. catumaxomab was started in December 2011 (4 consecutive i.p. infusions of catumaxomab on days 0, 3, 7 and 10 at increasing doses of 10, 20, 50 and 150 μg). Treatment was well tolerated without any side effects. Surveillance by CT scan in April, July and October 2012 showed a partial response of the pulmonary lesion and no paracentesis was necessary until January 2013 (Figure 3a and b). While the pulmonary lesion showed a further regression in size and could finally only be detected as a scar (Figure 4a), a CT scan in January 2013 showed a progressive peritoneal disease with ascites and a consecutive hydronephrosis III° of the right kidney (Figure 4b). As the patient still refused any further CTX we decided to restart i.p. treatment with catumaxomab in January 2013.

## Conclusions

Peritoneal carcinomatosis (PC) is one manifestation of metastatic CRC and is associated with a poor prognosis [3]. Few studies have been published describing the effectiveness of systemic chemotherapy in patients with PC. The results invariably show a disappointing response to systemic treatment and a poor prognosis compared to other metastatic sites [2,3]. CRC frequently overexpresses EpCAM [5]. Overexpression of EpCAM has been associated with dismal prognosis in other tumor entities, such as gallbladder cancer, ovarian cancer and pancreatic cancer [8-10]. Catumaxomab, the first anti-EpCAM antibody, was approved in 2009 for the treatment of malignant ascites in cancer patients with EpCAM positive tumors [6]. This prospective randomized phase II/III trial showed a significantly prolonged median puncture free survival with catumaxomab (46 days) compared to paracentesis alone (11 days) in the pooled population. Although the study was not powered to detect a difference in overall survival, OS showed a positive trend for the catumaxomab group and was significantly prolonged in patients with gastric cancer patients compared to paracentesis alone (71 versus 44 days;p = 0.0313). Interestingely, in our case report treatment with catumaxomab not only extended puncture free survival by 12 months but also caused a regression of the pulmonary metastasis suggesting a possible systemic effect. So far an extraperitoneal effect of catumaxomab has never been described in a patient with colorectal cancer and was reported for a patient with ovarian cancer and breast cancer [11,12]. A possible explanation for this systemic effect on tumor cells might be that catumaxomab is absorbed by lymphatics in the peritoneum and reaches the circulation. Low systemic catumaxomab levels (<1%) could be measured after i.p. infusion in nine out of thirteen patients with a high observed inter-individual variability [13]. Thus, an inverse correlation between tumor burden and systemic antibody bioavailability of catumaxomab was demonstrated in patients and in a defined mouse model [13]. The bioavailability of catumaxomab significantly declined in mice with higher tumor load. In the pivotal trial of Heiss et al. efficacy of catumaxomab was higher in patients without metastasis compared to patients with higher metastasis [6]. Therefore, in our patient a lower tumor burden might have resulted in higher plasma levels of catumaxomab. As blood samples have not been taken this determinant remains speculative. Furthermore an induction of anti-tumor specific T-lymphocytes that has been described after intraperitoneal administration of catumaxomab might be causative for its antiproliferative effect on distant metastasis [14]. Thus, in 5 out of 9 patients intraperitoneal administration of trifunctional antibodies such as catumaxomab induced a significant increase of tumor reactive CD4+/CD8+ T-lymphocytes with a prolonged survival. As catumaxomab is a nonhumanized chimeric antibody derived from mouse/rat IgG it is immunogenic when administered to humans. Thus, the development of human antimouse antibodies (HAMAs), which are associated with beneficial humoral effects and prolonged survival, could be detected in up to 95% of patients treated with catumaxomab [6]. In a *post hoc* analysis the correlation between the detection of HAMAS and clinical outcome was analyzed [15]. Patients who developed HAMAs after catumaxomab showed significant improvement in puncture-free survival, time to next puncture and OS [15]. Unaware of the impressive clinical outcome, no blood samples were taken from our patient in order to detect HAMAs. In the study of Heiss et al. the primary study objective was puncture free survival. Additionally the number of intraperitoneal tumor cells was counted before and after intraperitoneal administration of catumaxomab but no imaging method was used to assess the response to catumaxomab [6]. In the randomized phase IIa study by Baumann et al., response to catumaxomab in patients with platinum-resistant or –refractory epithelial ovarian cancer was assessed according to the Response Evaluation Criteria in Solid Tumors (RECIST) guidelines for the first time [16]. The study revealed that catumaxomab had only modest activity in platinum-resistant ovarian cancer with an overall response rate of 28% in the high- dose treatment arm (10,20,50 and 100 μg) compared to 5% in the low-dose group (10,10,10 and 10 μg) on days 0,3,7 and 10.

Taken together, our observation suggests that catumaxomab might have a relevant systemic effect on cancer cells and therefore might improve the prognosis of patients with EpCAM-positive tumors. Further investigations to prove and to further explain this systemic effect of catumaxomab are warranted.

### Consent

Written informed consent was obtained from the patient for publication of this case report and any accompanying images. A copy of the written consent is available for review by the editor of this journal.

## Abbreviations

CRC: Colorectal cancer; PC: Peritoneal carcinomatosis; i.p.: intraperitoneal; CTX: Chemotherapy; HAMAs: Human antimouse antibodies.

## Competing interests

The authors declare that they have no competing interests.

## Authors’ contributions

AB, FH and RG wrote the paper. TM assessed response by reviewing patients imaging studies. All authors read and approved the final manuscript.

## Pre-publication history

The pre-publication history for this paper can be accessed here:

http://www.biomedcentral.com/1471-2407/13/618/prepub
